# Reprogramming Methods Do Not Affect Gene Expression Profile of Human Induced Pluripotent Stem Cells

**DOI:** 10.3390/ijms18010206

**Published:** 2017-01-20

**Authors:** Marta Trevisan, Giovanna Desole, Giulia Costanzi, Enrico Lavezzo, Giorgio Palù, Luisa Barzon

**Affiliations:** Department of Molecular Medicine, University of Padova, 35121 Padova, Italy; marta.trevisan@unipd.it (M.T.); giovanna.desole@studenti.unipd.it (G.D.); giulia.costanzi.gc@gmail.com (G.C.); enrico.lavezzo@unipd.it (E.L.); giorgio.palu@unipd.it (G.P.)

**Keywords:** reprogramming method, induced pluripotent stem cells, retroviral vector, Sendai virus vector, episomal vector, gene expression

## Abstract

Induced pluripotent stem cells (iPSCs) are pluripotent cells derived from adult somatic cells. After the pioneering work by Yamanaka, who first generated iPSCs by retroviral transduction of four reprogramming factors, several alternative methods to obtain iPSCs have been developed in order to increase the yield and safety of the process. However, the question remains open on whether the different reprogramming methods can influence the pluripotency features of the derived lines. In this study, three different strategies, based on retroviral vectors, episomal vectors, and Sendai virus vectors, were applied to derive iPSCs from human fibroblasts. The reprogramming efficiency of the methods based on episomal and Sendai virus vectors was higher than that of the retroviral vector-based approach. All human iPSC clones derived with the different methods showed the typical features of pluripotent stem cells, including the expression of alkaline phosphatase and stemness maker genes, and could give rise to the three germ layer derivatives upon embryoid bodies assay. Microarray analysis confirmed the presence of typical stem cell gene expression profiles in all iPSC clones and did not identify any significant difference among reprogramming methods. In conclusion, the use of different reprogramming methods is equivalent and does not affect gene expression profile of the derived human iPSCs.

## 1. Introduction

Since Yamanaka’s breakthrough in 2006 [1], induced pluripotent stem cells (iPSCs) have revolutionized the stem cell field and have been applied to several branches of studies. iPSCs have been generated with several integrative [2,3,4,5,6,7] and non-integrative methods [8,9,10,11], the former exploiting viral vectors that integrate into the host cell genome and stably express the transgene, and the latter including any approach that enables the transient expression of the transgene in target cells. More recently, other approaches based on the use of modified mRNAs, proteins or small molecules are becoming established methods to reprogram somatic cells to a pluripotent state, despite being technically challenging or inefficient [10,12,13]. iPSCs, being pluripotent, held the great potential to give rise to virtually any tissue of the body and have been differentiated in numerous cell types (for a review see [14]). The first method applied to generate iPSCs was based on the use of retroviral vectors, relying on high efficiency due to the integration of the transgenes, low cost, and high repeatability. The downside of the retroviral vectors method is the risk of insertional mutagenesis making the generated iPSCs untranslatable to clinical practice [15]. Among non-integrative methods, Sendai virus-based vectors and episomal vectors remain the most commonly used, although modified mRNAs represent the gold standard approach for clinical practice [16]. Sendai virus-based vectors represent the safest viral-based approach to generate iPSCs since they are considered “zero footprint”, meaning that they do not enter the nucleus but remain cytoplasmic and are diluted from the cells with the physiological cell division [8]. The efficiency of generating iPSCs with Sendai virus vectors is among the highest [17] and these vectors are really versatile since they can transduce a variety of cell types due to their broad tropism [18,19]. Still, concerns remain about the use of a viral based system, its impact on reprogrammed cells and, moreover, the high cost that this technique implies. Episomal vectors, on the other hand, represent a very efficient and low cost way to generate virus-free iPSCs [17]. They have been used to generate iPSCs from skin fibroblasts and blood cells through nucleofection techniques and they allow replication of episomes into transfected cells in order to maintain high transgene expression levels and a transient effect [9]. iPSCs generated with any of these methods can be applied to study the physio-pathological basis of diseases, to perform high throughput drug-screening tests and, as a future goal to be achieved, to cure diseases through cell therapy and tissue engineering. Requirements for clinical application of human iPSCs (hiPSCs), besides safety, include standardization and reproducibility of laboratory protocols. In this regard, variability among hiPSC clones has been ascribed to the different reprogramming techniques, genetic background, batch of material used, and even laboratory personnel manipulating the cells [6,20,21,22,23]. In this study, a comparison of three different reprogramming methods was performed to test whether the reprogramming technique might have any impact on the gene expression profile of the derived clones. In order to avoid biases due to extrinsic factors, the reprogramming protocols were performed simultaneously starting from human neonatal foreskin BJ fibroblasts at the same passage, using the same batches of materials such as media and feeders and the same laboratory operator. We conclude that the different reprogramming methods are equivalent since they did not influence the hiPSC gene expression profiles, arguing that the differences previously reported [24,25] are ascribable to other factors, such as lab-to-lab technical biases and to the genetic background of the starting samples. 

## 2. Results

**Figure 1:**
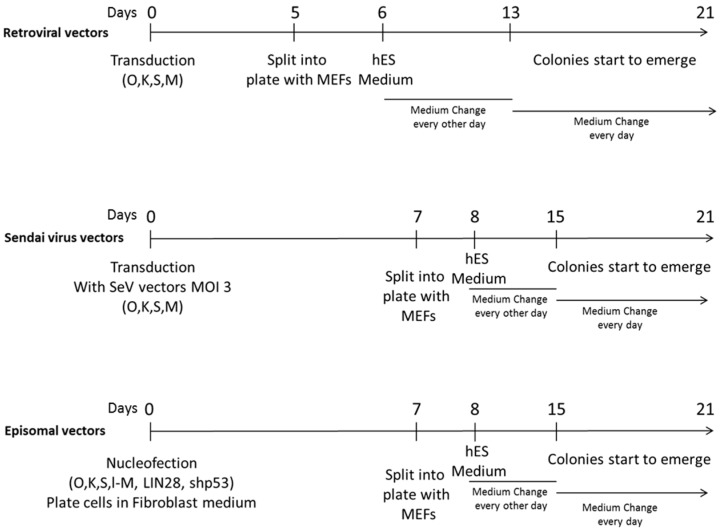
Schematic representation of the reprogramming protocols adopted to generate human induced pluripotent stem cells (hiPSCs) from somatic cells. Human neonatal foreskin BJ fibroblasts were reprogrammed exploiting three different protocols employing retroviral vectors, Sendai virus vectors, and episomal vectors, respectively. Briefly, for retroviral vector transduction, retroviral vectors were produced by transfecting HEK293T cells with pMIG plasmids (pMIG-SOX2, pMIG-OCT4, pMIG-KLF4, pMXS-cMYC) and the packaging plasmids pGag-Pol and p-VSV-G and used to transduce BJ fibroblasts. For Sendai virus vector transduction, BJ fibroblasts were transduced with Sendai virus vectors expressing the reprogramming factors OCT4 (O), KLF4 (K), SOX2 (S), cMYC (M) at a multiplicity of infection (MOI) of 3 each. For episomal vector transfection, BJ fibroblasts were nucleofected with three episomal plasmids expressing O, K, S, l-Myc, (l-M), LIN28 and a short interfering RNA to knock down p53 pathway (shp53). At 5 or 7 days post transduction or transfection, cells were seeded into MEFs and grown in hES Medium until colonies started to emerge (from 13 to 21 days after transduction/transfection). O: OCT4; K: KLF4; S: SOX2; M: c-Myc; MEFs: mouse embryonic fibroblasts; hES medium: human embryonic stem cells medium.

Reprogramming experiments using three different methods, i.e., based on retroviral vectors, Sendai virus vectors and episomal vectors, were conducted in parallel starting from BJ fibroblasts at early passages (passage 3), with the same batches of reagents, hoods and incubators and by the same operator. Reprogramming methods are outlined in Figure 1 and described in detail in the Methods section. hiPSC colonies started to appear around day 20 with all protocols; colonies were manually picked starting from day 25 post transduction/transfection and were grown under the same defined conditions and at the same passage rate for expansion and further characterization. 

**Figure 2:**
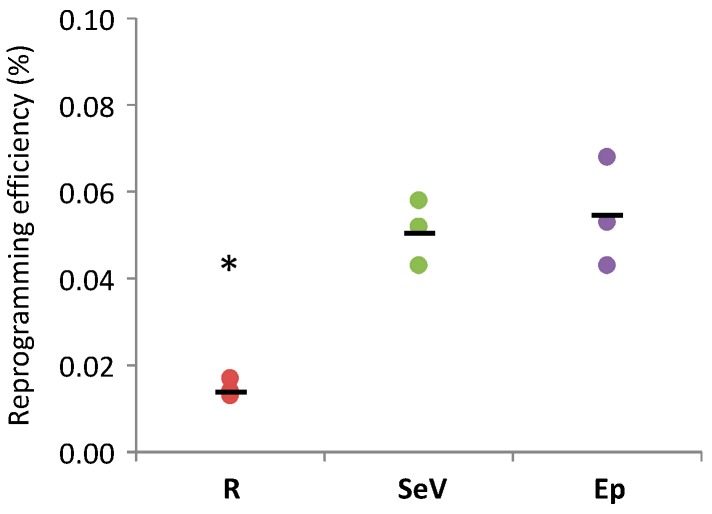
Comparison of the efficiency of reprogramming methods based on retroviral vectors (R), Sendai virus vectors (SeV), and episomal vectors (Ep). Reprogramming efficiency, calculated as the number of TRA1-60 positive human induced pluripotent stem cells (hiPSC) colonies obtained per starting number of transduced/transfected BJ cells, is represented by dots in the graph. Black bars indicate the mean efficiencies of reprogramming experiments; three experiments were conducted per each reprogramming method. * *p* < 0.05 R vs. SeV and R vs. Ep by Student’s *t*-test.

The efficiency of reprogramming was calculated as the number of Tra-1-60 positive hiPSC colonies over the starting number of cells and varied with the three different approaches (Figure 2). Among non-integrative methods, and in contrast with other data published in literature reporting a higher efficiency of Sendai virus vector than episomal vectors [17], the mean efficiency of the two approaches were comparable (about 0.05% of transduced cells for both Sendai virus vectors and episomal vectors). At variance, the retroviral vector-based method was the less efficient (i.e., about 0.01% of transduced cells). 

**Figure 3:**
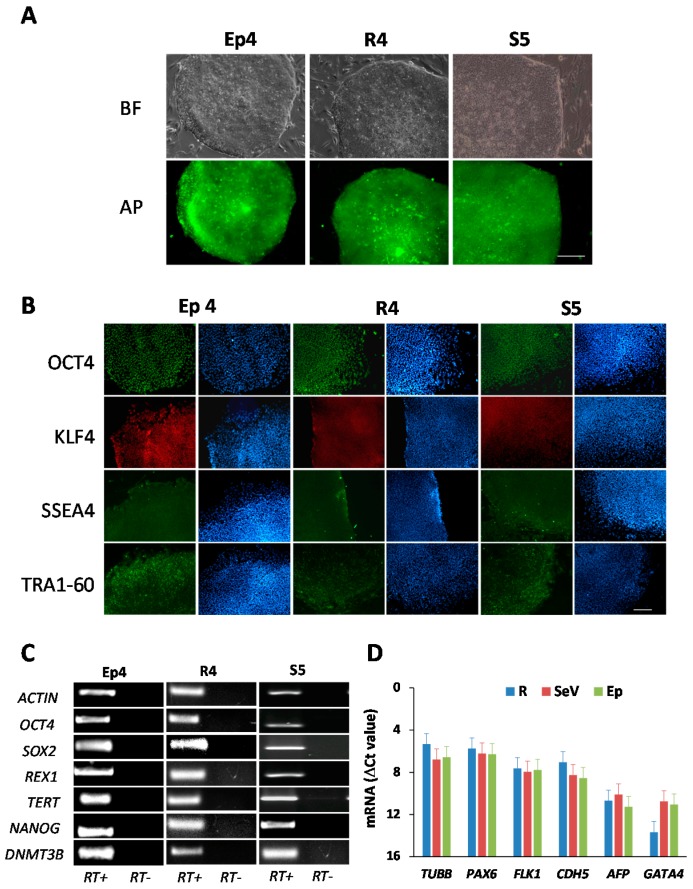
Characterization of human induced pluripotent stem cell (hiPSC) clones derived from human foreskin fibroblasts (BJ cells) by reprogramming with retroviral vectors (R), Sendai virus vectors (SeV), and episomal vectors (Ep). Data from a representative hiPSC clone for each reprogramming method are shown (R4, S5, Ep4). (**A**) Analysis of alkaline phosphatase (AP) expression in hiPSC clones; bright field (BF) images are also shown; (**B**) immunofluorescence staining of the pluripotent cell-specific markers OCT4, KLF4, SSEA4 and TRA1-60; DAPI in blue; (**C**) analysis of expression of the embryonic stem cell marker genes *OCT4*, *SOX2*, *REX1*, *TERT*, *NANOG*, *DNMT3B* by RT-PCR; (**D**) Embryoid Bodies (EBs) test: qRT-PCR analysis of differentiation markers of the three germ layers, i.e., ectoderm (*PAX6* and *TUBB*), endoderm (*AFP* and *GATA4*), and mesoderm (*FLK1* and *CDH5*), in EBs generated from iPSCs clones obtained with retroviral vector (R), Sendai virus vector (SeV), and episomal vector (Ep) transduction. Scale bars in panels (**A**,**B**) correspond to 200 µm.

In order to assess the pluripotency state of the derived hiPSC clones, 5 lines for each reprogramming method were further expanded and characterized. As shown in Figure 3, which displays only one representative clone per method, all the hiPSC lines expressed the undifferentiated state marker alkaline phosphatase (AP) detected by a live cell imaging assay (Figure 3A), the OCT4, KLF4, SSEA4 and TRA1-60 proteins detected by immunofluorescence assay (Figure 3B), and a panel of pluripotency genes, i.e., the *OCT4*, *SOX2*, *NANOG*, *DNMBT3*, *TERT* and *REX1* transcripts detected by RT-PCR (Figure 3C). In order to verify the propensity of the hiPSC lines to differentiate into the derivatives of the three germ layers, depicting their pluripotency state, we performed the embryoid bodies (EBs) test on all examined clones. This test, which is considered an in vitro surrogate for the teratoma formation test, helps to verify the potential of hiPSCs to be differentiated into virtually any cell type of the body and is performed by triggering a random differentiation of pluripotent cells by subtraction of both the adhesion stimuli and basic fibroblast growth factor (bFGF), the key factor that maintains cells in an undifferentiated state. As shown in Figure 3D, when tested by qRT-PCR for the expression of a panel of genes belonging to the ectodermal, endodermal and mesodermal layers, the EBs obtained from the different hiPSC clones expressed these markers, confirming their pluripotent state.

The gene expression profile in hiPSC clones obtained with the different protocols was further analyzed and compared by microarray analysis. A total of 14 hiPSC lines and BJ fibroblasts were evaluated. All the hiPSC lines analyzed were cultivated at the same defined condition and growth rate, and the passages at the time of RNA isolation were in the same range, from 5 to 9.

**Figure 4:**
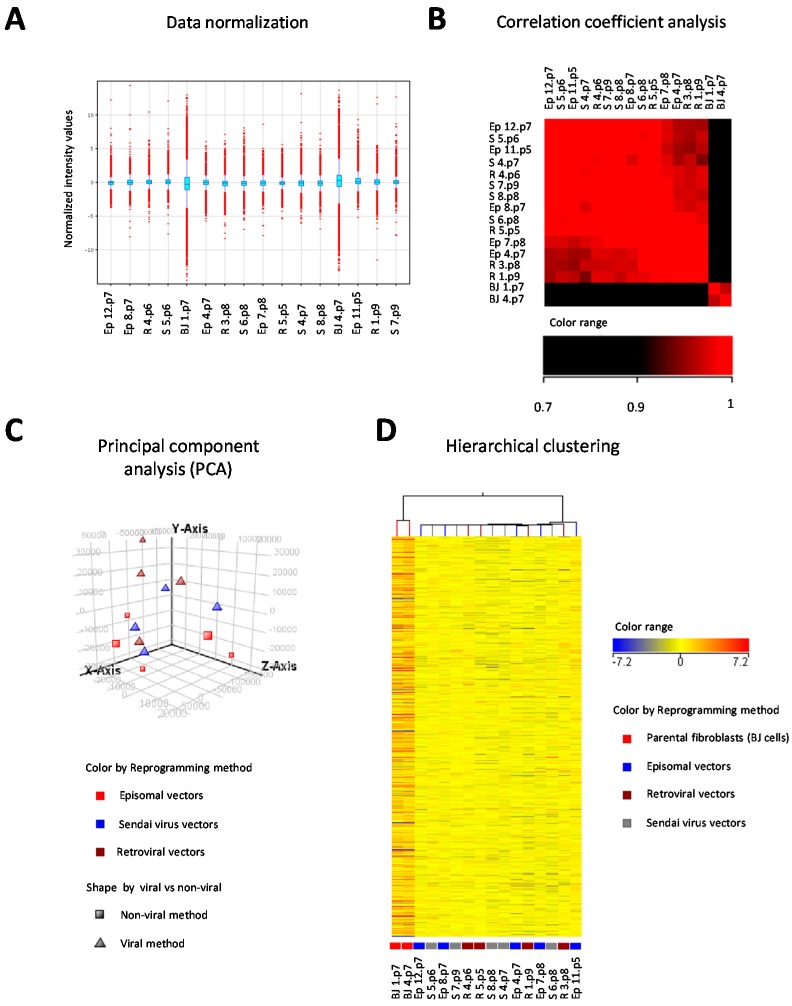
Global gene expression profile analysis by DNA microarrays of 14 human induced pluripotent stem cell (hiPSCs) lines reprogrammed with three different strategies, i.e., retroviral vectors (R), Sendai virus vectors (S) and episomal vectors (Ep), and parental BJ fibroblasts. (**A**) Distribution of normalized intensity values of each sample to the median of all samples is displayed in the box-whisker plot; thresholded to 1, shifted to 75.0 percentile; probes with intensity values beyond 1.5 times the interquartile range are shown in red; (**B**) correlation coefficient analysis of datasets; (**C**) principal component analysis (PCA) of datasets according to reprogramming methods and type of vectors (viral vs. non-viral); (**D**) unsupervised hierarchical clustering of samples according to intensity values and reprogramming protocols. A total of 41,093 entities was included in the analysis.

**Figure 5:**
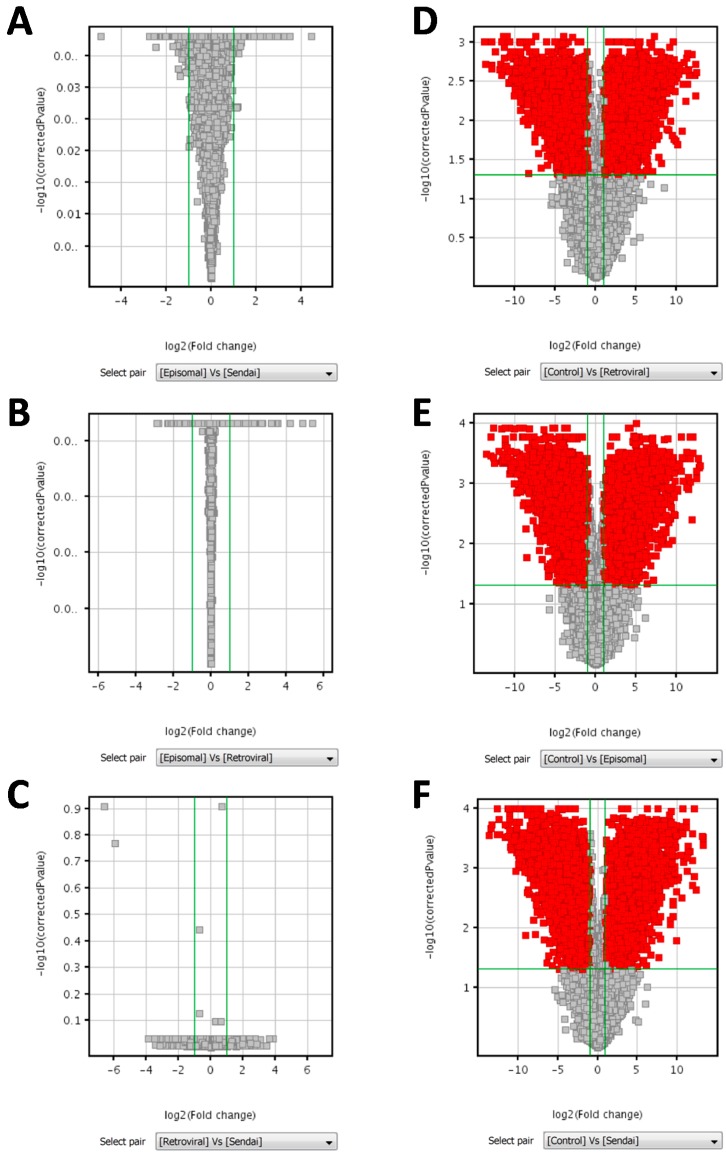
Volcano plots of pairwise comparisons between reprogramming methods and between hiPSC clones and parental BJ fibroblasts (Control). Groups were compared by unpaired Student’s *t*-test followed by Benjamini Hochberg false discovery rate (FDR) for multiple testing correction. Volcano plots of pairwise comparisons between hiPSC clones obtained by episomal vectors vs. Sendai virus vectors (**A**); episomal vectors vs. retroviral vectors (**B**); and retroviral vectors vs. Sendai virus vectors (**C**) displaying 0 entities out of 41,093, satisfying the *p*-value cut-off 0.05 and the fold change cut-off 2.0. Volcano plots of pairwise comparisons between hiPSC clones obtained by retroviral vectors (**D**); episomal vectors (**E**); and Sendai virus vectors (**F**) vs. parental BJ fibroblasts (Control) displaying, respectively, 11,191 entities, 12,230 entities, and 13,003 entities out of 41,093, satisfying the *p*-value cut-off 0.05 and the fold change cut-off 2.0. Green lines represent applied cut-off of Fold change (vertically) and of corrected *p*-value (horizontally). Red dots represent genes that were significantly up or down-regulated.

Microarray data were firstly normalized to the median, revealing a wider range of expression values in parental BJ fibroblast cells than in the derived hiPSC lines (Figure 4A). Correlation coefficient analysis (Figure 4B), principal component analysis (PCA) (Figure 4C), and unsupervised hierarchical clustering analysis (Figure 4D) indicated a clear segregation of all hiPSC clones from BJ cells, but no grouping of the hiPSC clones according to the reprogramming method adopted. In addition, analysis of data by using one way ANOVA test, Kruskal-Wallis test and Student *t* test did not identify any differentially expressed genes among hiPSC clones reprogrammed with different methods (i.e., methods based on episomal, Sendai virus, and retroviral vectors), between hiPSC clones reprogrammed with integrative (retroviral vectors) vs. non-integrative (Sendai virus and episomal vectors) methods, or between hiPSC clones reprogrammed with methods based on viral vectors (retrovirus and Sendai virus vectors) vs. non-viral vectors (episomal vectors). Volcano plots of pairwise comparisons between the different reprogramming methods are shown in Figure 5. 

At variance with this, comparison of the gene expression profile by ANOVA between hiPSC clones and parental BJ fibroblasts identified 15,726 differently expressed entities out of the 41,093 features analyzed (volcano plots of pairwise comparisons are shown in Figure 5). The differently expressed genes included those involved in pluripotency (e.g., *SOX2*, *OCT4*, *NANOG*, *LIN28*), which were upregulated in hiPSCs and those involved in the pathway of fibroblast growth (e.g., gene encoding for collagen proteins, laminin, and fibroblast growth factors), which were upregulated in fibroblasts (Table 1). Remarkably, the fold change expression values of these up or down regulated genes were highly similar among hiPSC clones obtained with the three reprogramming methods, as shown in Table 1 for a subset of genes, which were selected as markers of the undifferentiated and completely reprogrammed hiPSCs based on previously published data [17,21,26]. Gene Ontology analysis performed on the differently expressed genes demonstrated an association with DNA binding, transcription factors, regulation of gene expression, and morphogenesis processes.

## 3. Discussion

This study showed that three different reprogramming strategies adopted to obtain hiPSCs from somatic differentiated cells had comparable efficiency and generated hiPSCs with very similar gene expression profiles. Reprogramming methods included the use of retroviral vectors (i.e., integrative viral vector), Sendai virus vectors (i.e., non-integrative viral vector), and episomal vectors (non-integrative non-viral vector) to deliver the reprogramming genes. In order to evaluate the impact of the reprogramming methods on hiPSC gene expression profile, avoiding the noise of genetic background, tissue of origin and culture conditions, the reprogramming experiments were conducted starting from the same somatic cell line, and the BJ fibroblasts were used at the same early passage, with defined protocols, since it has been already demonstrated that even the choice of the feeder layer may influence the pattern of gene expression [27].

The efficiency of reprogramming ranged from 0.01% to 0.05% among methods (i.e., 0.05% for episomal and Sendai virus vectors and 0.01% for retroviral vectors). The efficiency of episomal vector reprogramming was about five times higher than previously reported [17,28]. At variance, retroviral vector reprogramming was less efficient than reported in the literature [29,30]. However, we cannot rule out that this lower efficiency might be due to the use of fresh non-titered batches of viral vectors for the transduction experiments. All the derived clones expressed at comparable levels the undifferentiated state marker alkaline phosphatase and a set of pluripotency factors and had the potential to be differentiated into all the derivatives of the three germ layers; ectoderm, mesoderm and endoderm.

Microarray analysis was performed on a subset of characterized hiPSC clones at early passages. Although hiPSCs acquire a more stable gene expression signature with sub-culturing [23,31,32], the choice of using such early passages was based on the fact that the cells might also acquire aberrations with the in vitro handling [33]. The results of the gene expression profile analysis showed that, regardless of the reprogramming approach, hiPSCs expressed the same set of genes at comparable levels and no differentially expressed genes could be detected by comparative analysis between hiPSC clones derived with viral and non-viral vectors or with integrative and non-integrative approaches. Our results are in agreement with a comprehensive study performed on a large subset of hiPSCs comparing non-integrating (i.e., Sendai virus vectors, episomal vectors and modified mRNA) and integrating (i.e., retroviral vectors, lentiviral vectors) reprogramming methods where the authors concluded that the subtle differences in gene expression levels detected among these reprogramming approaches were not method-specific and were confined to clone-specific signatures [17].

On the other hand, other authors have argued that even the sets of reprogramming factors harbor distinct DNA methylation aberrations in hiPSCs at the epigenome level, despite these being generated from the same parental cell type [34]. Notably, the use of a viral based system is also still a matter of debate when considering the stability of the hiPSC clones. Choi and colleagues recently demonstrated, exploiting a model of genetically matched hiPSC lines obtained by Sendai virus vector reprogramming of in vitro human embryonic stem (hES)-derived differentiated cells, that viral vector infection can significantly change the expression of a set of cellular genes [21]. At variance with this, in a similar model, Shutova and colleagues used a doxycycline inducible model to generate isogenic hiPSCs from somatic cells differentiated from hES cells and demonstrated that the subtle differences between the derived clones were rather laboratory-specific as the reprogramming process itself does not leave a common trace in isogenic hiPSC lines [23]. Of note, it is well known that the use of retroviral vectors might imply retrovirus-induced gene expression changes imputable to the integration of the provirus into gene, promoter or enhancer sequences or to chromatin silencing triggered by the provirus itself [15]. Indeed, we cannot rule out that the influence of retrovirus integration into one specific clone might be lost with the analysis performed in the present study. With the advance of the reprogramming techniques and the possibility to induce hiPSCs without the use of nucleic acids or with proteins it would be very interesting in the future to compare the gene expression profiles of the hiPSC clones generated with these methods.

In conclusion, the results of this study indicate that hiPSCs generated with the three different approaches possess similar expression profiles and that the reprogramming method does not have an impact on the cellular gene expression profile. It is conceivable that the differences at transcriptional levels previously reported for hiPSCs are ascribable more likely to lab-to-lab biases and to the genetic background of the starting material rather than to the reprogramming approach adopted [20,21,22]. The results of this work will be helpful in the choice of the reprogramming strategy to adopt when using hiPSCs suitable for drug screening or the clinical practice.

## 4. Materials and Methods

### 4.1. Cell Cultures

BJ fibroblasts, HEK-293T (both from ATCC, Manassas, VA, USA) and Mytomycin-C Inactivated Mouse Embryo Fibroblasts (MEFs), strain CF1 (Merck Millipore, Billerica, MA, USA) were cultured in Dulbecco Modified Medium, D-MEM, supplemented with 10% Fetal Bovine serum and 1% penicillin/streptomycin (all from Life Technologies, Carlsbad, CA, USA). hiPSCs culture: hiPSCs were grown either on MEFs feeder layer in human embryonic stem (hES) cells Medium (DMEM-F12, added with 20% Knockout Serum Replacement, 1% Non-essential Amminoacid, 2 mM GlutaMAX™, 1× 2-mercaptoethanol and 10 ng/mL basic-fibroblast growth factor (bFGF) (ORF Genetics, Kopavogur, Iceland) or on Matrigel™ (BD Biosciences, Franklin Lakes, NJ, USA) coated dishes in mTeSR-1™ medium (StemCell Technologies, Vancouver, BC, Canada).

### 4.2. hiPSCs Reprogramming

#### 4.2.1. Retroviral Vectors Reprogramming

To produce retroviral particles, HEK293T cells growing onto a 10 cm dish were transfected via lipofectamine 2000 (Life Technologies) with the packaging plasmid pGAG-POL, the envelope plasmid pVSV-G, and either the retroviral plasmids pMIG-SOX2 (#17226), pMIG-OCT4 (#17225), pMIG-KLF4 (#17227), or pMXS-cMYC (#13375), all a gift from George Daley [30] and achieved through Addgene (Cambridge, MA, USA). 48 and 72 h post-transfection, media were harvested, filtered through a 0.45 μm pore size filter and used to transduce 10^5^ BJ fibroblasts at passage 3 with polybrene 5 μg/mL (Sigma Aldrich, St. Louis, MO, USA). Five days post-transduction, fibroblasts were detached and seeded into a 10 cm-dish pre-coated with MEFs feeder layer. The next days, the medium was switched from D-MEM to hES medium and the cells were fed every other day for a further week. The medium was then changed every day until colonies started to emerge.

#### 4.2.2. Sendai Virus Vectors Reprogramming

SeV reprogramming was performed using the Cytotune^®^-iPS Sendai Reprogramming Kit (Life Technologies) following the manufacturer’s protocol. Briefly, 2.5 × 10^5^ BJ fibroblasts at passage 3 were transduced with each of the four viruses at a multiplicity of infection (MOI) of 3 and the medium was changed every other day. On day 7 post transduction, 1.25 × 10^5^ cells were plated onto a 10-cm dish previously coated with MEFs feeder layer. The day after, the medium was switched to hES medium and the cells were fed every other day for a week before switching to the daily feeding. Once the colonies emerged, they were picked by mechanical dissection and transferred to a fresh feeder.

#### 4.2.3. Episomal Vectors Reprogramming

For episomal vector reprogramming, sub-confluent BJ fibroblasts at passage 3 were detached by tripsinization and 5 × 10^5^ cells were nucleofected with the program for human dermal fibroblast NHDF using nucleofection solution P2 (Nucleofector 4D, Amaxa, Lonza, Basel, Switzerland) with 1 µg of each plasmid, pCXLE-hOCT3/4-shp53-F, pCXLE-hSK, pCXLE-hUL (a gift from Shinya Yamanaka, Addgene plasmid #27077, 27078 and 27080; [9]). Upon nucleofection, cells were readily plated onto a 0.1% gelatin (EmbryoMax™ Millipore) coated 10 cm dish in fibroblast medium supplemented with Rock Inhibitor 10 μM (Sigma Aldrich) and the medium was changed every 3 days. On day 7 post-nucleofection, fibroblasts were enzymatically detached with trypsin (Life Technologies) and seeded onto 10 cm dishes covered with MEFs-feeder cells. On the next day the medium was switched to hES medium and changed every other day until hiPSC-like colonies started to appear.

### 4.3. Tra-160 and Alkaline Phosphatase-Live Assay

For Tra-160 and Alkaline Phosphatase live staining, respectively, to reprogramming plates and hiPSCs growing on 24 well plates were added 1× TRA-1-60 Mouse anti-human mAb, (AlexaFluor^®^ 488 Conjugate Kit for Live Cell Imaging, Life Technologies) or 1× AP Live stain solution (Life Technologies). After an incubation of 30 min at 37 °C, the cells were washed twice with DMEM/F12 and the fluorescence was observed in a Leica microscope (Leica, DFC 420C, Wetzlar, Germany).

### 4.4. Immunofluorescence Analysis

hiPSCs growing for 5 days on 24 well plates were washed three times with DPBS (Life Technologies), fixed in PFA 4% for 20 min at RT, permeabilized with 0.1% Triton X-100 in PBS for 15 min at RT and blocked overnight at 4 °C in 4% BSA/PBS (Sigma Aldrich). The primary antibodies were incubated 1 h at RT, followed by incubation with the secondary antibodies. The following primary antibodies were used: goat anti-Oct3/4 (Santa Cruz Biotechnology Inc., Dallas, TX, USA), mouse anti-SSEA4 (Abcam, Cambridge, UK), mouse anti-TRA-1-60 (Abcam), mouse anti-KLF4 (Merck Millipore). Upon incubation with the appropriate secondary antibodies (AlexaFluor^®^ 488 Donkey anti-goat (Thermo Fisher Scientific, Whaltham, MA, USA), H&L (FITC) and Goat anti-Mouse, both from Abcam) cells were stained with DAPI (Life Technologies), fluorescence was observed in a Leica microscope and images were taken using LAS V3.8 software (Leica). 

### 4.5. RNA Extraction

hiPSC clones at the same passages were grown for 5 days on Matrigel coated plates with mTesR1 and detached with Accutase (Life Technologies). Total RNA was purified from harvested cells using RNeasy mini Kit (Qiagen, Venlo, Limburg, The Netherlands) following manufacturer’s instructions. The RNA concentration and purity were determined loading 1 µL of the sample into the NanoDrop 1000 Spectrophotometer (Thermo Fisher Scientific) through measurement of the A260/280 ratio. For microarrays analysis, confirmation of the RNA quality was performed using a 2100 Bioanalyzer (Agilent Technologies, Santa Clara, CA, USA) following the preparation protocol provided by Agilent RNA 6000 Nano kit (Agilent Technologies).

### 4.6. Pluripotency Marker Expression by RT-PCR 

Total RNA purified from the hiPSC clones was treated with TURBO DNase (Ambion, Thermo Fisher Scientific) in order to avoid the contamination of genomic DNA. 1 µg of total RNA was reverse transcribed to cDNA with Random Hexamers using a MuLV Reverse Transcriptase (all from Applied Biosystems, Thermo Fisher Scientific). RT-PCR was then performed to evaluate the expression of pluripotency genes *DNMT3B*, *OCT4*, *TERT*, *NANOG*, *REX1* and *SOX2*.

### 4.7. Embryoid Bodies Test

Embryoid bodies (EBs) formation was performed by detaching the hiPSCs growing on MEFs feeder layer with Collagenase IV (Invitrogen, Thermo Fisher Scientific) and plating them into Corning^®^ Ultra-Low attachment multi-well plate (Corning, NY, USA) in hES medium depleted of bFGF. The medium was changed every three days for a week, after which the bodies were transferred into a 0.1% gelatin pre-coated 6-well plate with DMEM 10% FBS 1% penicillin–streptomycin, 2 mM Glutamax (all from Life Technologies). After 7 days of growth in adhesion, cells were harvested by trypsinization and total RNA was collected and converted to cDNA as previously described. Quantitative RT-PCR analysis was performed to amplify with the SYBR (Thermo Fisher Sicentific) green chemistry genes expressed in the ectodermal (*TUBB* and *PAX6*), mesodermal (*FLK1*, *CDH5*) and endodermal layer (*AFP*, *GATA4*). A 2^−ΔΔ*C*t^ analysis was performed using the *GAPDH* gene as an endogenous housekeeping gene used for normalization.

### 4.8. Gene Expression Microarray Analysis

Gene expression profiling was carried out using SurePrint G3 human GE microarrays (Agilent Technologies, Santa Clara, CA, USA) according to the manufacturer’s protocol using 1 µg of total RNA as starting material. Briefly, preparation and labeling of RNA samples were performed following the Quick Amp Labeling Kit procedure (Agilent Technologies). Each RNA sample was labeled with fluorescent dye Cyanine-3 (cRNA) and purified using RNeasy mini kit (Qiagen); cRNAs were quantified with a Nanodrop spectrophotometer (Thermo Fisher Scientific). Hybridization of cRNA samples to SurePrint G3 human GE microarray slides (Agilent Technologies) was performed for 17 h at 56 °C according to the manufacturer’s protocol. Microarrays scanning were performed using an Agilent Microarray Scanner (Agilent Technologies). Raw data are available as Supplementary Materials.

### 4.9. Statistical Analysis

Statistical analysis of the data was performed using Gene Spring GX 11.5.1 software (Agilent Technologies). Data pre-processing included quality control analysis of samples, setting threshold raw signals to 1.0, log base 2 transformation, and normalization to median values of all entities. Exploratory data analysis by correlation coefficient analysis, Principal Component Analysis (PCA), and unsupervised hierarchical clustering was performed to individuate distances/similarities and gradients/patterns in the gene expression profile among the analyzed hiPSC clones and BJ cells. In order to identify differentially expressed genes between hiPSCs and parental BJ cells and among iPSC reprogramming methods, the one way ANOVA test and the non-parametric Kruskal Wallis test, both followed by Benjamini Hochberg False Discovery Rate (FDR) correction (*p*-value < 0.05), were performed. Furthermore, Student *t* test for unpaired data, followed by Benjamini-Hochberg FDR correction (*p*-value < 0.05), was applied for pairwise comparison of the reprogramming method. Differentially expressed genes between hiPSC and BJ cells were further analyzed to extract their functional information. For each gene, the corresponding Gene Ontology (GO) annotations was extracted from the Gene Ontology Annotation database (GOA, available at: http://geneontology.org/); then, GO annotations extracted from upregulated and downregulated genes were clustered separately using the GO Class function provided by the Argot2.5 webserver [35], resulting in sets of functional categories that summarize the differences observed in the gene expression levels.

## Figures and Tables

**Table 1:** Genes differentially expressed between human induced pluripotent stem cells (hiPSCs) generated with three reprogramming methods and parental human neonatal foreskin BJ fibroblasts.

Gene Symbol	Probe Name	Regulation	LogFc Ep vs. BJ	LogFc R vs. BJ	LogFc S vs. BJ
*RIN2*	A_24_P305570	down	−6.10	−5.79	−6.33
*RIN2*	A_23_P91379	down	−5.33	−5.00	−6.02
*RIN2*	A_23_P120572	down	−4.10	−4.90	−4.84
*LMNA*	A_23_P34835	down	−4.32	−4.45	−4.02
*LMNA*	A_24_P162718	down	−4.32	−4.60	−4.01
*EMILIN1*	A_23_P165848	down	−5.35	−5.28	−5.39
*TMEM173*	A_23_P61371	down	−7.80	−7.84	−7.93
*COL3A1*	A_23_P142533	down	−9.50	−8.48	−9.97
*COL3A1*	A_24_P402242	down	−8.65	−7.81	−9.61
*COL3A1*	A_24_P935491	down	−9.04	−8.09	−9.46
*FAP*	A_23_P56746	down	−12.49	−12.37	−13.23
*FGF5*	A_24_P401855	down	−6.43	−6.25	−6.53
*FGF5*	A_23_P212800	down	−8.96	−8.97	−8.98
*GBP3*	A_23_P51487	down	−10.10	−10.26	−10.38
*GBP3*	A_24_P370702	down	−8.60	−9.34	−9.31
*LYST*	A_23_P354074	down	−7.05	−6.59	−6.24
*ANPEP*	A_23_P88626	down	−8.20	−8.59	−8.20
*POU5F1*	A_24_P144601	up	11.66	11.67	11.40
*POU5F1*	A_23_P59138	up	11.29	11.52	11.36
*POU5F1*	A_32_P132563	up	10.57	10.70	10.40
*POU5F1*	A_24_P214841	up	11.28	11.34	11.11
*CDH1*	A_23_P206359	up	11.04	11.43	11.12
*LEFTY1*	A_23_P160336	up	5.49	5.85	5.15
*TDGF1*	A_32_P135985	up	7.98	8.29	7.98
*TDGF1*	A_23_P366376	up	12.70	12.94	12.67
*SALL4*	A_23_P109072	up	7.48	7.13	7.62
*RAB25*	A_23_P115091	up	7.64	7.32	7.58
*PTPN6*	A_23_P162486	up	3.80	3.56	3.91
*EPHA1*	A_23_P157333	up	8.59	8.39	8.41
*SCNN1A*	A_23_P128323	up	5.64	5.34	5.57
*SOX2*	A_23_P401055	up	13.09	12.99	13.71
*SOX2*	A_24_P379969	up	9.93	9.85	10.22
*NANOG*	A_23_P204640	up	9.19	9.78	9.28
*TERT*	A_23_P110851	up	5.14	5.49	5.01
*DNMT3B*	A_23_P28953	up	7.89	8.30	8.00

Logarithmic Fold Change (LogFc) variation; Ep: episomal vector-based reprogramming; R: retroviral vector-based reprogramming; S: Sendai virus vector-based reprogramming.

## Supplementary Materials

Supplementary materials can be found at www.mdpi.com/1422-0067/18/1/206/s1.
